# Porosity Distribution in Single Cone Root Canal Fillings Performed by Operators with Different Clinical Experience: A microCT Assessment

**DOI:** 10.3390/jcm10122569

**Published:** 2021-06-10

**Authors:** Saulius Drukteinis, Goda Bilvinaite, Paulius Tusas, Hagay Shemesh, Vytaute Peciuliene

**Affiliations:** 1 Institute of Dentistry Faculty of Medicine Vilnius University, Zalgirio 115, LT-08217 Vilnius, Lithuania; goda.bilvinaite@gmail.com (G.B.); paulius.tusas@gmail.com (P.T.); vytaute.peciuliene@mf.vu.lt (V.P.); 2 Academic Centre for Dentistry Amsterdam (ACTA), Gustav Mahlerlaan 3044, 1081 LA Amsterdam, The Netherlands; H.Shemesh@acta.nl

**Keywords:** single cone, BioRoot RCS, general dental practitioner, endodontist, micro-computed tomography, porosity

## Abstract

The objective of this study was to assess the porosity distribution of BioRoot RCS/single gutta-percha point root-canal fillings performed by a general dental practitioner and endodontist. Fourteen plastic models of maxillary premolars with two roots were selected and shaped with HyFlex EDM instruments to a size 40/0.04 taper and randomly divided into two experimental groups. A total of 14 canals in each group were obturated by two different operators with one HyFlex EDM size 40 gutta-percha point and BioRoot RCS sealer. The specimens were scanned with a high-resolution micro-computed tomography scanner, and the porosity of the fillings at the coronal, middle, and apical thirds of the root canals was qualified and quantified. The differences between groups and root-canal thirds were compared using Mann–Whitney, Friedman, and Wilcoxon tests with the significance level set at *p* < 0.05. The highest number of pores was observed in the apical third of root-canal fillings in both groups; however, the porosity distribution between the two groups was not significantly different in the apical and middle thirds (*p* > 0.05). Statistically significant differences were determined only in the coronal third (*p* < 0.05). The predominance of open pores was detected in all root-canal thirds and groups, with no significant differences in the number of open pores between the coronal and middle thirds (*p* > 0.05).

## 1. Introduction

The quality of root-canal obturation is usually assessed using periapical radiographs to evaluate the length and homogeneity of the filling. It has been considered, that properly obturated root canal should be filled up to the full working length (WL) with a conically shaped and homogeneous filling material [1]. However, the two-dimensional (2D) radiographic image does not always provide sufficient information about the real homogeneity, tightness, and sealability of the filling. It is considered that root-canal filling can ensure the best long-term success of endodontic treatment if the filling material seals the entire root canal up to the apical constriction [2]. The root-canal fillings prevent recontamination of the root-canal system by fluids with microorganisms and their metabolites from the oral cavity [3]. It also creates unfavorable conditions for the survival of microorganisms, remaining after chemomechanical root-canal preparation, and disrupts their penetration into periapical tissues [2]. It has been demonstrated that the homogeneity, tightness, and sealability of the fillings highly depend on the pores inside the root-canal filling material [4]. The two types of pores can be detected: open or external, and closed or internal [5]. The open pores form between the filling material and the root-canal walls and therefore create a through-and-through “network” of the pores providing the excellent conditions for the microleakage and negatively affecting the outcome of endodontic treatment [2,6]. Meanwhile, the internal pores are entrapped inside filling material and do not have any substantial adverse clinical impact [5].

To avoid or minimize the formation of the pores inside the root-canal fillings, various materials and obturation techniques were proposed and are used nowadays [7]. The most popular solid root-canal filling material is gutta-percha, which has good biocompatibility, plasticity, and radiopacity, is dimensionally stable, and easily inserted or removed from the root canal if needed [8]. However, this material does not adhere to the root-canal wall dentin; therefore, a sealer is needed to ensure a hermetical seal of the root canals [3]. The most popular root-canal obturation method is cold lateral compaction of the gutta-percha [7]. However, despite its popularity, the porosity of the laterally compacted gutta-percha/sealer fillings is relatively high in comparison to the other obturation techniques, especially in curved root canals [9]. Similar results have been shown by Gupta et al. (2015), demonstrating the worse homogeneity and a higher porosity of the laterally condensed gutta-percha fillings in comparison to thermoplastic obturation methods [10]. The comparable findings were published by Keles et al. (2014) revealing significantly less porosity of the vertically compacted thermoplastic gutta-percha in comparison to the cold lateral compaction [11]. However, the scientific data on the advantage of any specific obturation technique on the outcome of endodontic treatment is still controversial and debatable [5,12].

Recently, the single cone (SC) root-canal obturation or “cold hydraulic obturation” technique was introduced as a simplified method for root-canal obturation [13]. A single tapered gutta-percha point in conjunction with a newly developed flowable hydraulic calcium silicate-based sealers are used with this technique [13,14]. Due to the physicochemical properties of the hydraulic calcium silicate-based sealers, they can be used as fillers instead of the sealers, allowing the clinician to increase the material’s volume inside the root canal [15]. The tapered gutta-percha points are used to increase the hydraulic pressure inside the root canal and improve the sealer’s distribution as well as to make the SC filling removable if the retreatment is indicated [13,16]. The previous investigations have shown that the porosity distribution in SC fillings is comparable to that in other obturation techniques, or even less [14,17,18]. Furthermore, the SC technique is very simple to use for the clinicians [19], but at the same time demonstrates a high clinical success rates in both primary endodontic treatment and endodontic retreatment [20,21].

The majority of studies reporting the success rates of endodontic procedures are based on treatments performed by highly trained and experienced professionals—endodontists (ED) or postgraduate students [12,20]. The same applies for experimental studies, as laboratory work is usually performed by highly experienced operators too [10,11,17]. However, when the endodontic treatment procedures are performed by the differently skilled clinicians, the quality of the endodontic treatment differs significantly [22,23]. Bajawi et al. (2018) have found that the quality of root-canal obturation performed by ED was significantly better in comparison to general dental practitioners (GDP) [24]. The same was observed by other investigators, demonstrating better clinical outcomes when endodontic treatment was performed by ED in comparison to GDP [25,26]. It should be highlighted that these studies usually involved cases in which root canals were obturated using cold lateral or warm vertical compaction of the gutta-percha. However, there is little information how the clinical experience can impact the quality of the root-canal obturation using the SC technique in conjunction with hydraulic calcium silicate sealer.

Microcomputed tomographic (µCT) imaging has recently become the technique of choice to assess the quality of root-canal cleaning, shaping, and obturation in vitro [14,27]. The technique ensures the possibility of characterizing filling materials, and quantifying and qualifying the pores, voids, and gaps inside the material or at the material/root-canal wall interface [5,6]. The main advantage of the µCT method is its nondestructiveness, repeatability, and high accuracy [17]. There are few published articles, evaluating the porosity of SC root-canal fillings [14,17,28]. However, there is no data about porosity distribution in the SC fillings, performed by operators with different competencies and clinical experience. Therefore, this in vitro study aimed to determine the porosity type and distribution in SC root-canal fillings using µCT imaging. The null hypothesis tested was that the clinical experience of the operator has no significant impact on the quality of the root-canal obturation.

## 2. Materials and Methods

### 2.1. Specimen Preparation

Fourteen standardized plastic models of maxillary premolars (DRSK, Hassleholm, Sweden) with pre-opened endodontic access, two separate roots and Type I canal configuration, according to Weine classification, were used in this experiment. A size 10 K-file (Dentsply Maillefer, Ballaiques, Switzerland) was inserted inside the root canal to determine the WL 1 mm short of the apical foramen. All root canals were subsequently enlarged with HyFlex EDM instruments (Coltene, Langenau, Germany) to the full WL. Instruments were driven at the rotation speed of 400 rpm and the torque of 2.5 Ncm using X-Smart (Dentsply Sirona, Ballaiques, Switzerland) endodontic motor. The following sequence of the files was used: 10/0.05, 20/0.05, 25/~, 40/0.04. After the use of each instrument, the root canals were irrigated with 5 mL 3% sodium hypochlorite (Ultradent Products Inc., South Jordan, UT, USA) using disposable syringes and 29-G NaviTip needles (Ultradent Products Inc., South Jordan, UT, USA). At the end of preparation, 5 mL of 17% ethylenediaminetetraacetic acid (Ultradent Products Inc., South Jordan, UT, USA) was used to irrigate the root canals for 2 min before they were dried with size 40/0.04 taper paper points (Coltene, Langenau, Germany). All procedures were performed by the same operator. 

### 2.2. Root-Canal Obturation

After instrumentation plastic models were fixed into prefabricated A-silicone (3M Express, 3M ESPE, Seefeld, Germany) blocks up to the cemento-enamel junction to ensure the blindness of the root-canal filling procedure, and randomly assigned into two groups (*n* = 7), according to the operator performing root-canal obturation: GDP and ED. Both clinicians had over fifteen years of the clinical experience in the field of their competencies and were familiar with a SC obturation technique using hydraulic calcium silicate-based sealer.

Fourteen canals in each group were obturated with one HyFlex EDM size 40.04 gutta-percha point (Coltene, Langenau, Germany) and BioRoot RCS (Septodont, Saint-Maur-des-Fosses, France) sealer. The sealer was mixed according to the manufacturer’s instructions. The pre-fitted master gutta-percha point was coated with a thin layer of sealer and gently inserted into the root canal to cover the walls. The procedure was repeated twice to ensure the required amount of the sealer. On the last—third—time, the gutta-percha point was recoated with the sealer again and slowly inserted into the root canal to the full WL. After the cut of the gutta-percha point at the orifice level, the endodontic access cavities were isolated with Cavit™-W (3M ESPE, Seefeld, Germany) and immersed into 37 °C temperature water for one week to allow the filling material to set completely before further analysis.

### 2.3. µCT Scans and Analysis

All specimens were scanned with a high-resolution µCT scanner SkyScan 1272 (Bruker, Kontich, Belgium) at 90 kV and 111 µA using a 0.5 mm aluminum and 0.038 mm copper filter, 0.2° rotation step, 10 µm isotropic resolution, and 1350 ms exposure time. The reconstruction of images was performed using NRecon v.1.7.1.0 software (Bruker, Kontich, Belgium) with a beam-hardening correction of 20% and ring-artefact reduction factor of 3. 

The CTAn v.1.14.4.1 software (Bruker, Kontich, Belgium) was used to analyze the reconstructed images. After the selection of root-canal contours and the segmentation of grayscale images, the custom-processing tool was used to process binary images and quantify the volume of pores (VVol), filling material (FVol), closed pores (CPVol), and open pores (OPVol). The total volume of the root canal (CVol) was determined by the formula: CVol = VVol + FVol. The percentage volume of closed pores (%CPVol) and open pores (%OPVol) was calculated as follows:

%CPVol = CPVol/CVol × 100,

%OPVol = OPVol/CVol × 100.

The quantitative analysis was performed separately for the coronal, middle, and apical thirds at intervals of 3 mm. The last apical 1 mm of the root length was excluded. The evaluation of the images was accomplished by one individual, who had no information regarding the experimental groups and their specimens. The CTVol v.2.2.3.0 software (Bruker, Kontich, Belgium) was used to create three-dimensional (3D) models and visualize the fillings and porosity distribution.

### 2.4. Statistical Analysis

SPSS 25.0 software (SPSS Inc., Chicago, IL, USA) was used for statistical analysis. The data revealed a non-normal distribution, validated with the Shapiro–Wilk test. Therefore, the non-parametric Mann–Whitney test was selected to transform the percentages of open and closed pores to ranks and determine the differences between two experimental groups. The Friedman and Wilcoxon tests were used to detect the differences between thirds in the same group. A *p*-value < 0.05 was considered as statistically significant. 

## 3. Results

**Figure 1:**
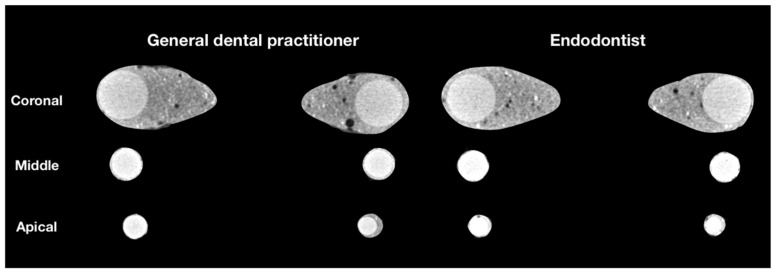
Representative cross-sectional 2D images at the coronal, middle and apical thirds of random samples of GDP and ED groups, demonstrating the porosity distribution of the fillings.

The µCT analysis revealed pores of various sizes between the filling material and root-canal walls as well as inside the filling material (Figure 1). 

The mean ranks of open and closed pores are summarized in Table 1, where the higher mean rank in the same column refers to the higher porosity of root-canal fillings. The distribution of closed pores demonstrated statistically significant differences between the two experimental groups in the coronal (*p* < 0.001) and middle (*p* = 0.036) thirds, while the distribution of open pores significantly differed only in the coronal third (*p* = 0.046). The apical thirds of root-canal fillings exhibited the highest number of open pores. However, no significant differences were observed between groups in the apical third by comparing the number of both open (*p* = 0.225) and closed (*p* = 0.052) pores.

Table 2 presents the results of pairwise comparison of thirds in the same group. Pairwise comparisons were applied after obtaining significantly different results by comparing the porosity distribution between all thirds together (*p* < 0.05). Regarding the results of pairwise comparisons, the number of open pores remained similar between the coronal and middle thirds in both groups (*p* > 0.05). Furthermore, no statistically significant differences were detected between the middle and apical thirds by comparing the distribution of closed pores in the GDP group (*p* = 0.208).

**Figure 2:**
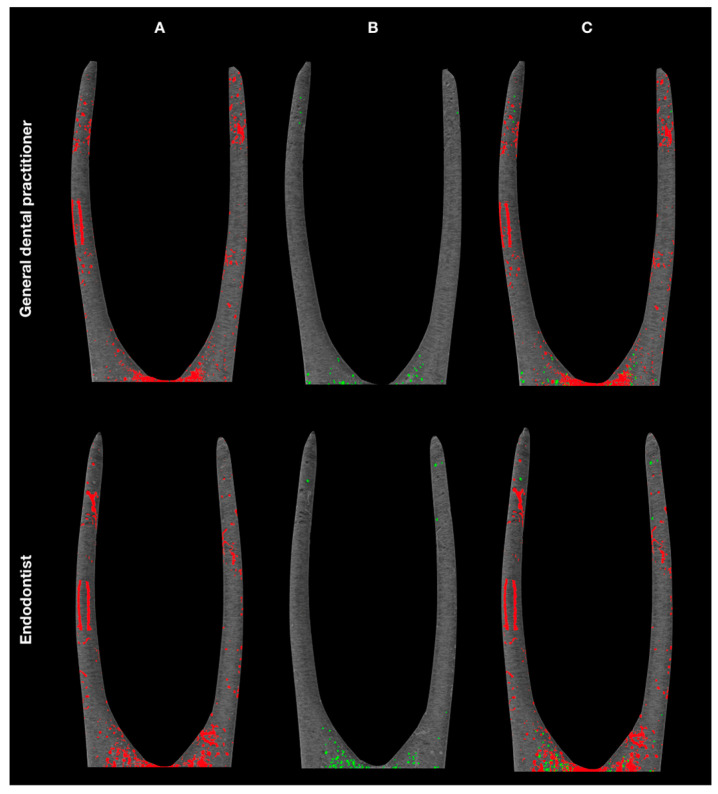
3D reconstructions demonstrate the distribution of open (**A**, red) and closed (**B**, green) pores, and overall porosity (**C**, superimposed picture) of root-canal fillings in GDP and ED groups.

Figure 2 represents the 3D reconstructions of the obturated root canals, demonstrating that open pores were the dominant type of porosity in both groups.

## 4. Discussion

The quality of root-canal obturation is clinically assessed, evaluating the length and homogeneity of the fillings in periapical radiographs. However, it should be highlighted that the visible pores in the fillings on the radiographs represent just 2D reality and do not provide 3D information. Therefore, the accurate evaluation of the porosity of the fillings in clinical practice using radiographs is relatively limited, but at the same time, it is the only method available for the clinicians. Previous investigations demonstrate that the percentage of pores in root-canal fillings detectable by volumetric 3D µCT analysis is much higher in comparison to the 2D findings [6,12,22,29]. However, there is no clear evidence to determine which level of the porosity is critical and can have a direct negative impact on the outcome of endodontic treatment [12,14,30]. Moreover, it has been concluded that the overall porosity is not the critical indicator and that the number of the open pores plays a crucial role, as it is related to the increased risk of microleakage [30]. The µCT is nowadays considered as the technique of choice for the assessment of the quality of root-canal obturation in in vitro experiments [6,17]. The technique—due to its non-destructiveness, repeatability, and precision—demonstrates its superiority over the other methods, such as bacterial, glucose, radioactive isotope, dye penetration tests, or SEM investigations [31,32]. In this study, the porosity distribution in SC fillings performed by operators with different clinical experience was assessed, and the type of pores was identified using a high-resolution µCT scanner. The standardized plastic 3D models of the premolars were used for µCT assessment, making the samples uniform and comparable, the sample size more homogenized. The advantages of the use of the standardized models have been already discussed previously [17]; however, some drawbacks exist. The main disadvantage of the plastic blocks as compared to natural teeth is the difference between plastic dentine. Dentinal tubules, intratubular moisture, irregular shape, and diameter/volume of root canals are the factors that could possibly affect the hydration and behavior of the hydraulic calcium silicate-based sealers and their physical properties, including the porosity [17,33]. However, it should be highlighted that the primary aim of this investigation was to assess the 3D quality of the SC fillings performed by GDP and ED, using the clinical experience factor as the determinant for the comparison.

It has been demonstrated previously that the clinical experience of the operator has a substantial impact on the quality of the endodontic procedures and clinical outcome [12,34]. It has been shown that the lowest root-canal obturation quality was observed among undergraduate dental students, and gradually improved over a period of time after graduation and acquisition of clinical experience [34,35]. Finally, the best results and the highest success rates were achieved when endodontic treatment was performed by an ED [36]. However, it should be mentioned that these studies used conventional root-canal cleaning and shaping procedures, and cold lateral or warm vertical compaction of the gutta-percha. Interestingly, Kharouf et al. (2019) demonstrated that the porosity distribution in root-canal fillings performed by undergraduate students significantly dropped when the SC technique was used instead of lateral compaction of gutta-percha [34]. However, there is no data available about the quality of SC fillings performed by a GDP and its comparison to the quality of fillings made by an ED. Therefore, this study investigated the porosity distribution in SC fillings performed by GDP and ED—two operators with different competencies and clinical experience. However, due to the lack of published studies, our results cannot be compared with previous investigations.

The impact of the root-canal filling technique on the porosity distribution is extremely controversial [6,17]. The lateral and vertical gutta-percha compaction techniques are the most popular and widely investigated by in vitro and in vivo studies [3,7]. However, the results did not demonstrate any significant differences in the porosity distribution among these obturation techniques [5,8]. Moreover, the clinical success and outcomes of endodontic treatment remained comparable when these techniques were used [12]. Meanwhile, the SC fillings have demonstrated lower or equal porosity in comparison to cold lateral, warm vertical, or hybrid obturation techniques [6,14,37]. However, the data about SC fillings using the modern fourth or fifth generation of the hydraulic calcium silicate-based sealers are still limited [38].

In this study, the sealability and porosity of the SC fillings were comparatively evaluated by means of µCT imaging in the coronal, middle, and apical thirds of the root canals of both experimental groups. The previous research demonstrated the substantial clinical impact of the tight and pore-free filling of the apical third of the root canal, as it can adversely affect the outcome rates of the endodontic treatment [5,7,12]. The results of our study have shown that there were no significant differences in the porosity distribution between the fillings performed by the two operators in the apical thirds. However, in the middle thirds of the root canals, considerable differences were observed only in closed pores. These findings indicate that both operators were able to fill the apical and middle thirds of the root canals equally effectively—considering that the closed pores, which are entrapped into the sealer mass, do not negatively affect the long-term sealability and microleakage of the fillings [6,17,39]. Only in the coronal third of the root canals the overall porosity of the fillings, including open and closed pores, was significantly different between the GDP and ED groups. Our results are in agreement with previous in vitro investigations, demonstrating that the predominant type of the pores inside SC fillings is open or external pores, and that the most porous third of the fillings is the apical third [6,14]. However, it should be mentioned that these studies were evaluating the fillings performed by experienced operators (ED), and there is no data on the porosity distribution in SC fillings when the obturation procedure was accomplished by a GDP. 

A recently published retrospective clinical investigations demonstrated the quite high success rates of the outcome of endodontic treatment or retreatment, when SC technique in conjunction with hydraulic calcium silicate-based sealers was used [20,21]. However, the obturation procedures in these studies were performed by EDs or postgraduate endodontic students [21,35]. Nevertheless, there are no observations and clinical outcome results at the level of GDP. Based on the results from this study it can be hypothesized that if GDP and ED were able to achieve comparable results, there is a potential possibility that the quality of the root-canal obturation using the SC technique at the level of general dental clinical practice can be comparable to the ED. However, widespread and well-controlled clinical trials are needed to assess and confirm this hypothesis.

## 5. Conclusions

Within the limitations of this in vitro study, it can be concluded that operators with different competencies and clinical experience were not able to ensure pore-free root-canal fillings when the SC obturation technique in conjunction with hydraulic calcium silicate-based sealer was used. The open pores were the dominant type of pores in both experimental groups and all three thirds, with the highest porosity in the apical third of root-canal fillings. The quality and homogeneity of SC root-canal fillings remained comparable between GDP and ED groups in the apical and middle thirds, while the only significant differences were observed in the coronal third.

## Figures and Tables

**Table 1:** Mean ranks of open and closed pores in the coronal, middle, and apical thirds.

Group	*N*	Coronal Third		Middle Third		Apical Third	
		Open Pores	Closed Pores	Open Pores	Closed Pores	Open Pores	Closed Pores
GDP	14	16.38 ^A^	18.67 ^A^	14.88 ^A^	16.25 ^A^	14.92 ^A^	10.05 ^A^
ED	14	10.65 ^B^	7.77 ^B^	10.57 ^A^	9.89 ^B^	11.23 ^A^	15.37 ^A^

Different superscript letters in the same column indicate significant difference between groups (Mann–Whitney test; *p* < 0.05).

**Table 2:** *p*-values from pairwise comparison of thirds in the same group.

Group	Thirds	Open Pores	Closed Pores
GDP	Coronal–Middle	0.390 *	0.002
	Coronal–Apical	0.034	0.041
	Middle–Apical	0.010	0.208 *
ED	Coronal–Middle	0.197 *	0.005
	Coronal–Apical	0.002	0.001
	Middle–Apical	0.001	0.001

* Indicates a non-significant difference (pairwise Wilcoxon test; *p* > 0.05).

## Data Availability

Data is contained within the article.
